# Tryptophan-related dipeptides in fermented dairy products suppress microglial activation and prevent cognitive decline

**DOI:** 10.18632/aging.101909

**Published:** 2019-05-23

**Authors:** Yasuhisa Ano, Yuka Yoshino, Toshiko Kutsukake, Rena Ohya, Takafumi Fukuda, Kazuyuki Uchida, Akihiko Takashima, Hiroyuki Nakayama

**Affiliations:** 1 Laboratory of Veterinary Pathology, Graduate School of Agricultural and Life Sciences, the University of Tokyo, Tokyo 113-8657, Japan; 2 Research Laboratories for Health Science & Food Technologies, Kirin Company Ltd., Yokohama-shi, Kanagawa 236-0004, Japan; 3 Faculty of Science, Gakushuin University, Toshima-ku, Tokyo 171-8588, Japan

**Keywords:** aging, Alzheimer’s disease, inflammation, cognitive function, microglia, dairy products

## Abstract

The rapid growth in aging populations has made prevention of age-related memory decline and dementia a high priority. Several epidemiological and clinical studies have concluded that fermented dairy products can help prevent cognitive decline; furthermore, intake of Camembert cheese prevents microglial inflammation and Alzheimer’s pathology in mouse models. To elucidate the molecular mechanisms underlying the preventive effects of fermented dairy products, we screened peptides from digested milk protein for their potential to regulate the activation of microglia. We identified dipeptides of tryptophan–tyrosine (WY) and tryptophan–methionine that suppressed the microglial inflammatory response and enhanced the phagocytosis of amyloid-β (Aβ). Various fermented dairy products and food materials contain the WY peptide. Orally administered WY peptide was smoothly absorbed into blood, delivered to the brain, and improved the cognitive decline induced by lipopolysaccharide via the suppression of inflammation. Intake of the WY peptide prevented microglial inflammation, hippocampal long-term potential deficit, and memory impairment in aged mice. In an Alzheimer’s model using 5×FAD mice, intake of the WY peptide also suppressed microglial inflammation and accumulation of Aβ, which improved cognitive decline. The identified dipeptides regulating microglial activity could potentially be used to prevent cognitive decline and dementia related to inflammation.

## Introduction

With the rapid growth in the proportions of older population worldwide, cognitive decline and dementia are becoming an increasing burden on not only patients and their families but also national healthcare systems. Lack of a disease therapy for dementia has resulted in preventive approaches receiving increasing attention. In Alzheimer’s disease, amyloid-β (Aβ) and phosphorylated Tau become aggregated and are deposited as senile plaques and neurofibrillary tangles (NFTs), respectively, in older people [1,2]. Recent studies have reported that deposition of Aβ and phosphorylated Tau induces inflammation in the brain and exacerbates neurological deficits and cognitive decline [3–5]. Inflammation in the brain is regulated by microglia. Microglia plays a key role in maintaining the neuroenvironment by removing pathogens, waste  products, and old synapses via phagocytosis and by promoting synapse extension [6]. Proliferation and activation of microglia in the brain around senile plaques and NFTs are prominent features of Alzheimer’s disease [4]. Activated microglia are known to be associated with disease progression. Regulating microglial function has therefore been attracting increasing attention for therapy and prevention of dementia, including Alzheimer’s disease [7,8].

A recent epidemiological study results suggest that consumption of certain dairy products can reduce the risk of cognitive decline in elderly individuals and prevent Alzheimer’s disease. Camfield et al. suggested that some ingredients might be beneficial for promoting healthy brain function during aging [9]. Crichton et al. revealed that individuals who consumed low-fat dairy products, including yogurt and cheese, once a week had a higher cognitive function of memory recall and social functioning than did those who did not [10]. Ozawa et al. surveyed >1000 dementia-free 60- to 79-year-old Japanese subjects living in a local community to investigate their dietary patterns and any potential association with reduced risk of dementia [11,12]. The researchers concluded that inclusion of milk or fermented dairy products in the diet reduced the risk of dementia in the general Japanese population. In addition, we previously demonstrated that intake of a dairy product fermented with *Penicillium candidum*, i.e., Camembert cheese, suppressed Aβ deposition and activation of microglia in the brain of the Alzheimer’s disease model (5×FAD) mice [13]. These results suggested that some ingredients, such as peptides generated during fermentation, suppress inflammation in the brain and prevent cognitive decline. However, the underlying mechanism remains to be completely elucidated [14].

Some reports have shown that peptides have neuroprotective activities and improve cognitive function. Previous studies have demonstrated that cerebrolysin, a mixture of peptides derived from the brains of pigs, showed protective effects on cognitive decline and Alzheimer’s disease. In preclinical studies, cerebrolysin reduced inflammation, lowered cognitive impairment, and reduced the plaques and tangles present in patients with Alzheimer’s disease [15–18]. In a study involving patients with schizophrenia, cerebrolysin-treatment improved cognition and memory [19]. In another study of older adults with memory loss, a peptide preparation derived from cerebrolysin improved memory performance [20,21]. These study results encouraged us to explore for peptides derived from milk proteins that may prevent Alzheimer’s disease pathology.

Our own previous experiments have suggested that some ingredients in Camembert cheese suppressed microglial inflammation and Alzheimer’s pathology. Therefore, in the present study, we investigated the peptides from casein protein, predominant protein in Camembert cheese, which are regulating the activation of microglia in the brain, for their effects on cognitive decline in aged mice and Alzheimer’s disease model mice.

## RESULTS

### Identification of peptides that suppressed the activation of microglia and enhanced Aβ phagocytosis

**Figure 1:**
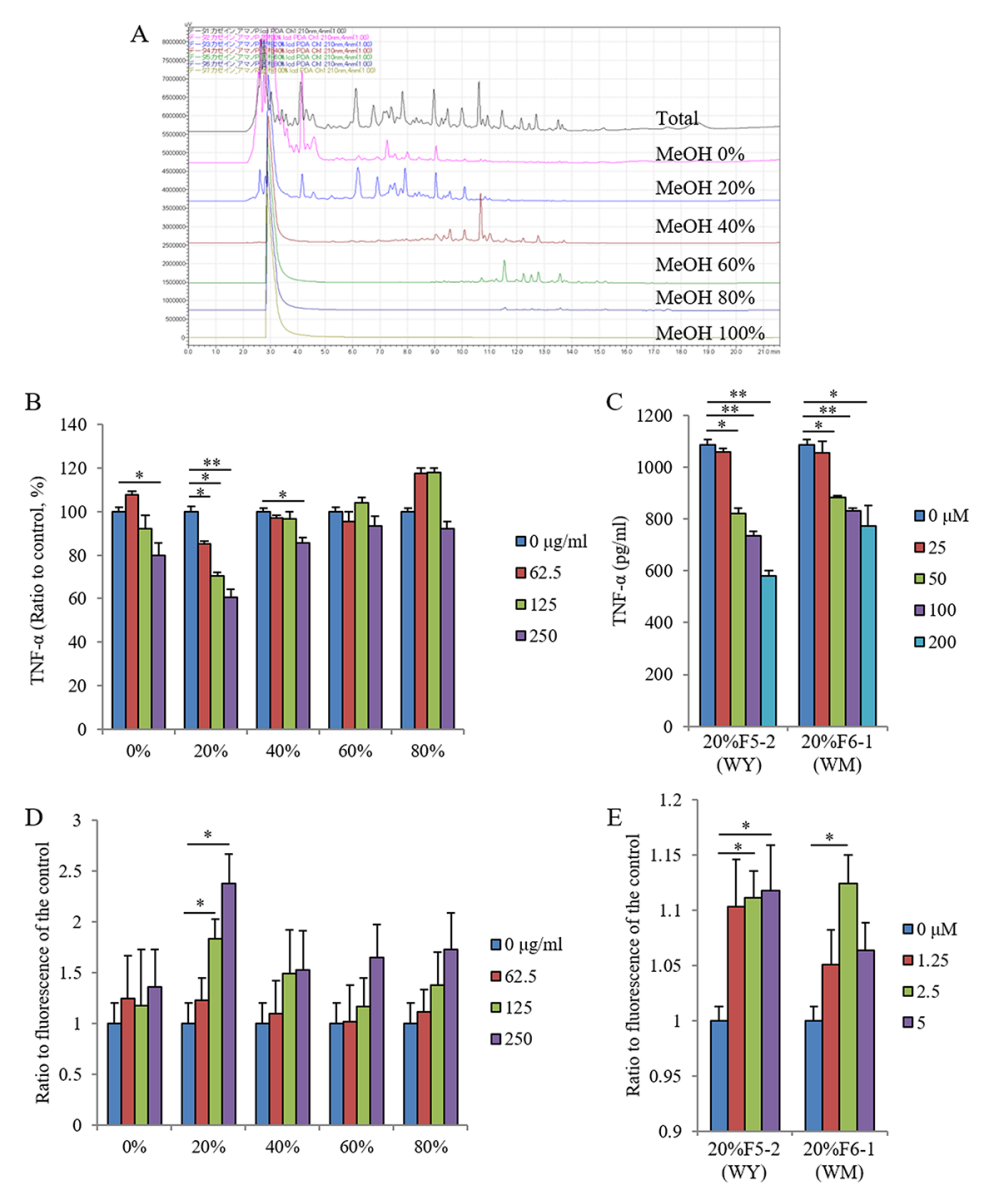
**Effects of the fractions and peptides on inflammatory response and phagocytic activity in microglia.** Casein protein was digested by using enzyme and fractionated by using a solid-phase column with 0%, 20%, 40%, 60%, and 80% methanol. (**A**) Chromatograph of each fraction using HPLC. (**B, C**) Effects of each fraction of methanol elution and dipeptides of WY and WM on inflammatory responses determined by using CD11b-positive primary microglia, respectively. The TNF-α production in supernatant of microglia culture pretreated with each fraction and dipeptides and then treated with LPS. (**D, E**) Effects of each fraction and the dipeptides on phagocytosis of Aβ determined by using CD11b-positive primary microglia, respectively. The intracellular fluorescence of Aβ-FAM in the microglia treated with each sample. Data are means ± SEM of 3–5 wells per sample. The *p*-values were calculated by using one-way ANOVA followed by Dunnett test. *p < 0.05 and **p < 0.01.

The enzyme digestions of casein protein showed both the suppression of TNF-α production after lipopolysaccharide (LPS) stimulation and the enhancement of Aβ-FAM phagocytosis in primary microglia derived from newborn mice. Therefore, the agents responsible for these effects were characterized. Figure 1A shows the solid-phase separations eluted with 20%, 40%, 60%, 80%, and 100% methanol. The fraction eluted with 20% methanol suppressed TNF-α production in response to LPS stimulation (Figure 1B), and purification and Edman sequences identified WY and WM peptides as effect-causing components (Figure 1C).

The fraction with 20% methanol increased Aβ phagocytosis (Figure 1D), and the purified fractions of WY and WM peptides increased Aβ phagocytosis (Figure 1E). These results indicate that WY and WM dipeptides, which contain tryptophan at the N-terminal, regulate microglial activation. Because the activities of the WY peptide for microglial regulation were higher than those of the WM peptide (Figure 1C and E), we used the WY peptide for the rest of the experiments.

### Blood–brain barrier penetration of the WY peptide

To understand the pharmacokinetics of the WY peptide, Trp-[carboxyl-^14^C]Tyr (^14^C-WY) peptide (Table 1A) was administered to rats, and radioactivity was measured in the blood at various time points. The ^14^C-WY peptide was incorporated into blood as early as 2 min after oral administration, and its concentration reached a maximum at 2.67 h (Table 1B). The tissue distribution of radioactivity at 2 h after oral administration showed that the tissue-to-plasma concentration ratio was 0.23 in the hippocampus and 0.24 in the cerebral cortex (Table 1C). There were no differences in the distribution of radioactivity among the different brain regions. These results indicate that the WY peptide or its metabolites penetrated into the brain and directly affected the activation of microglia and cognitive function.

**Figure:**
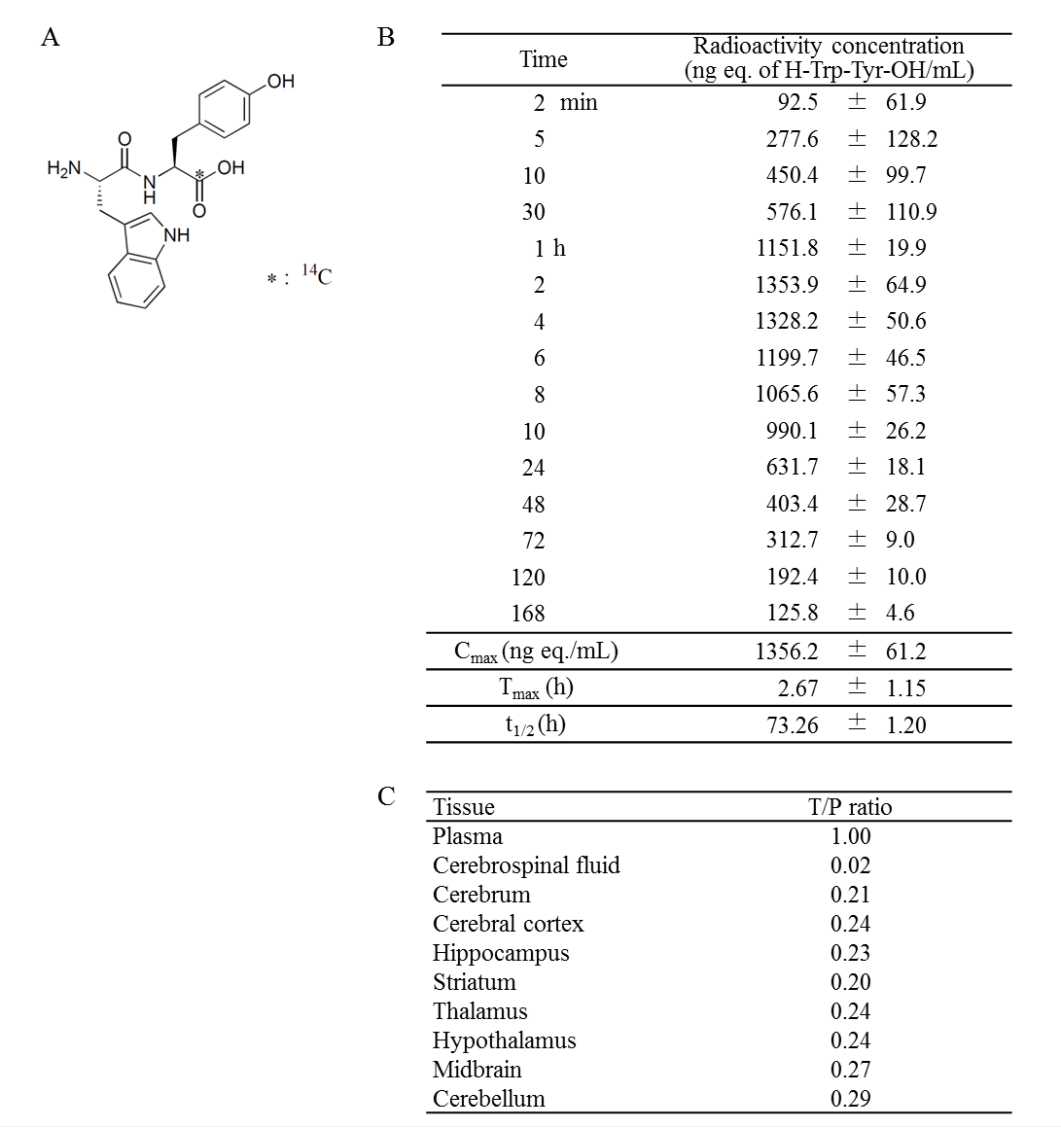
**Table 1. Pharmacokinetics of the ^14^C-WY peptide.** (**A**) Chemical structure of the ^14^C-WY peptide. (**B**) Radioactivity of blood samples treated with the ^14^C-WY peptide. (**C**) Distributions of radioactivity in each organ treated with the ^14^C-WY peptide

### Improvement of the WY peptide on memory impairment induced by inflammation

**Figure 2:**
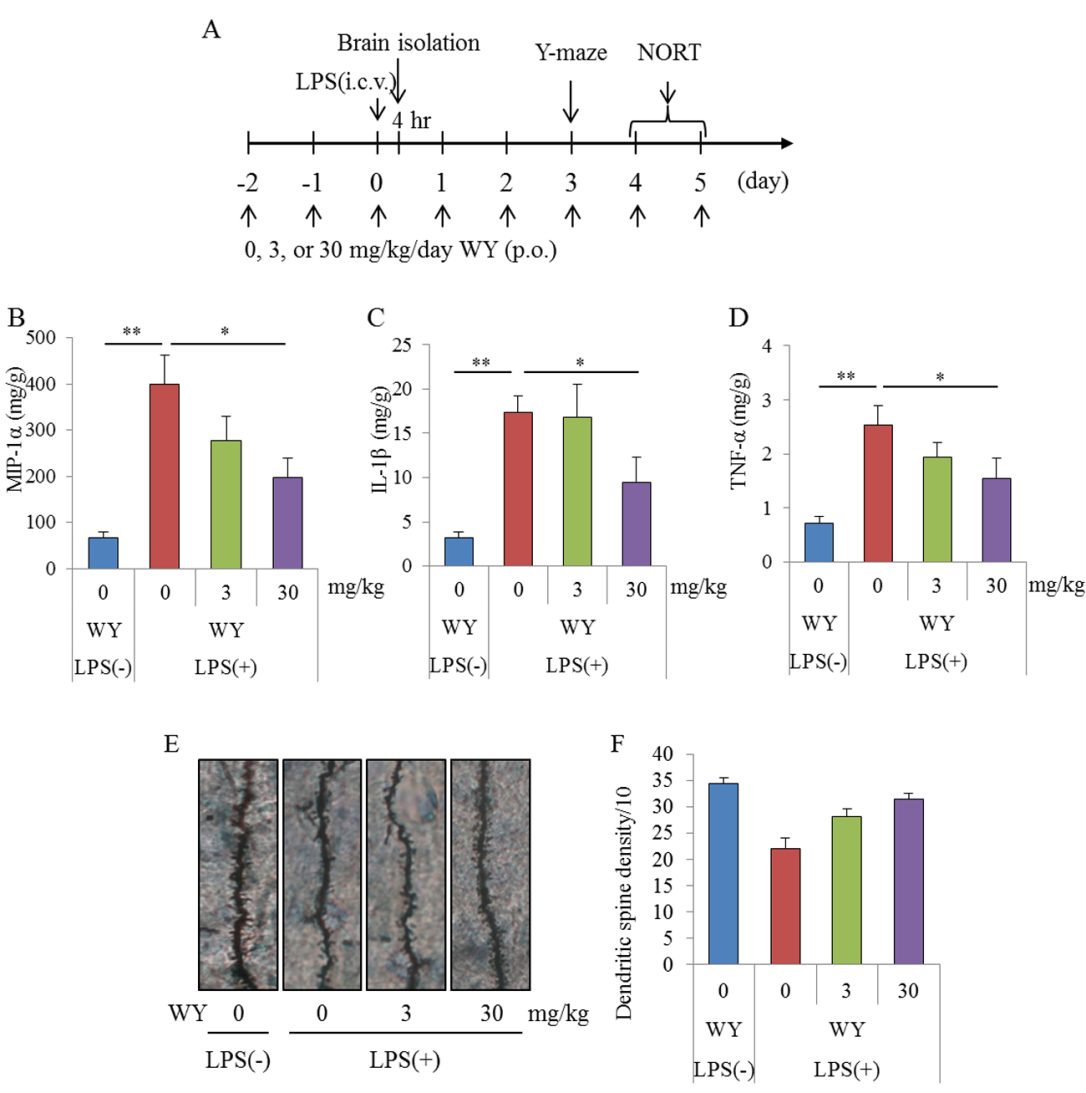
**Effects of WY peptides on inflammation induced by LPS.** Mice intracerebroventricularly injected with 5 μg of LPS at day 0 were orally administered 0, 3, or 30 mg/kg WY peptide from day −2 to day 0. (**A**) Scheme of this experiment. (**B, C, D**) The levels of MIP-1α, IL-1β, and TNF-α in the hippocampus of mice at 4 h after LPS injection, respectively. The hemisphere of the brain was subjected to Golgi staining. (**E**) Representative photomicrographs of Golgi-stained neurons in the CA1 of the hippocampus. (**F**) Number of dendritic spines per 10 μm in the CA1. Data are the mean ± SEM of 10 mice per group. *p*-values shown in the graph were calculated by performing Student *t*-test (LPS [−] vs. [+] at 0 mg/kg WY peptide) and one-way ANOVA followed by Dunnett test. *p < 0.05 and **p < 0.01.

To evaluate the effects of the WY peptide on cognitive decline induced by brain inflammation, mice injected intracerebroventricularly with LPS were orally administered the WY peptide and subjected to a Y-maze test and novel-object recognition task (NORT) (Figure 2A). The productions of TNF-α, MIP-1α, and IL-1β in the hippocampi of mice administered LPS injections were significantly increased compared with those in sham mice, and the cytokine productions in mice administered 30 mg/kg WY peptide were significantly reduced compared with the control mice (Figures 2B, C, and D, respectively). Next, to evaluate the effects of the WY peptide on neural dendrites in LPS-treated mice, dendrites were analyzed by Golgi staining. LPS injection to the brain significantly reduced the number of apical dendrites of pyramidal neurons and spines along those dendrites in the CA1 region of the hippocampus relative to mice receiving control treatment, but this effect of LPS was significantly prevented by prior treatment with the WY peptide (Figures 2E and F). These results indicate that administration of the WY peptide reduced LPS-induced inflammation in the brain and the concomitant dendritic atrophy of pyramidal neurons in the hippocampus.

**Figure 3:**
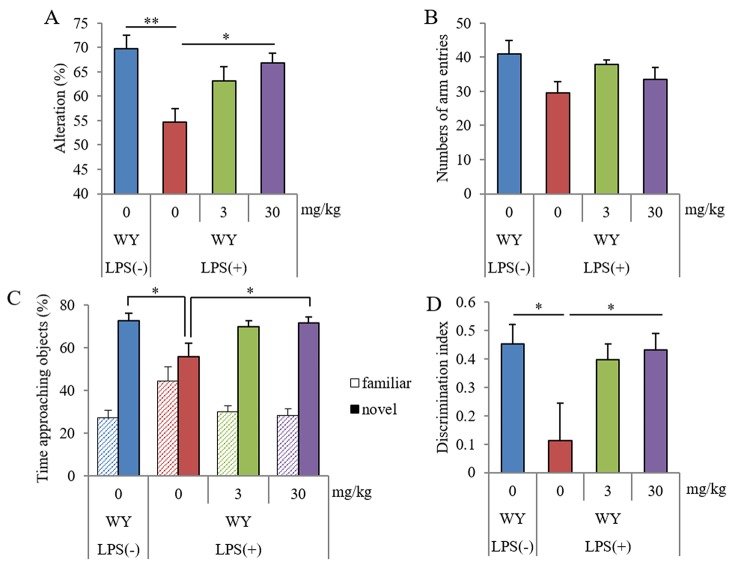
**Effects of WY peptides on memory impairment induced by LPS.** Mice intracerebroventricularly injected with 1.0 mg/kg of LPS at day 0 were orally administered 0, 3, or 30 mg/kg WY peptide from day −2 day to day 5. Mice were subject to the Y-maze test on day 3 and to NORT on days 4 and 5. (**A, B**) Spontaneous alterations and arm entries during 8 min of exploring in Y-maze to evaluate spatial memory. (**C**) Time spent exploring novel and familiar objects during 5 min of re-exploration as a percentage of the total time spent exploring objects to evaluate episodic memory. (**D**) Discrimination index [time spent with object A − time spent with object B] / [total time exploring both objects]. Data are the mean ± SEM of 10 mice per group. *p*-values shown in the graph were calculated by performing Student *t*-test (LPS [−] vs. [+] at 0 mg/kg WY peptide) and one-way ANOVA followed by Dunnett test. *p < 0.05 and **p < 0.01.

The Y-maze test showed that the spontaneous alterations in mice administered LPS injections were lower than those in sham mice, and the alterations in mice orally administered 30 mg/kg WY peptide were higher than those in control mice (Figure 3A). The arm entries of Y-maze were not significantly changed among the groups (Figure 3B). The NORT showed that the ratio to the approaching time for a novel object in LPS-injected mice was lower than that in sham mice, and the ratio in mice orally administered 30 mg/kg WY peptide was higher than that in the control mice (Figure 3C). The discrimination index was in accordance with the ratio to the approaching time (Figure 3D). Total distance was not changed among the groups (data not shown). These results showed that oral administration of the WY peptide improved LPS-induced spatial and object recognition memory impairment.

### Preventive effects of the WY peptide on cognitive decline in aged mice

**Figure 4:**
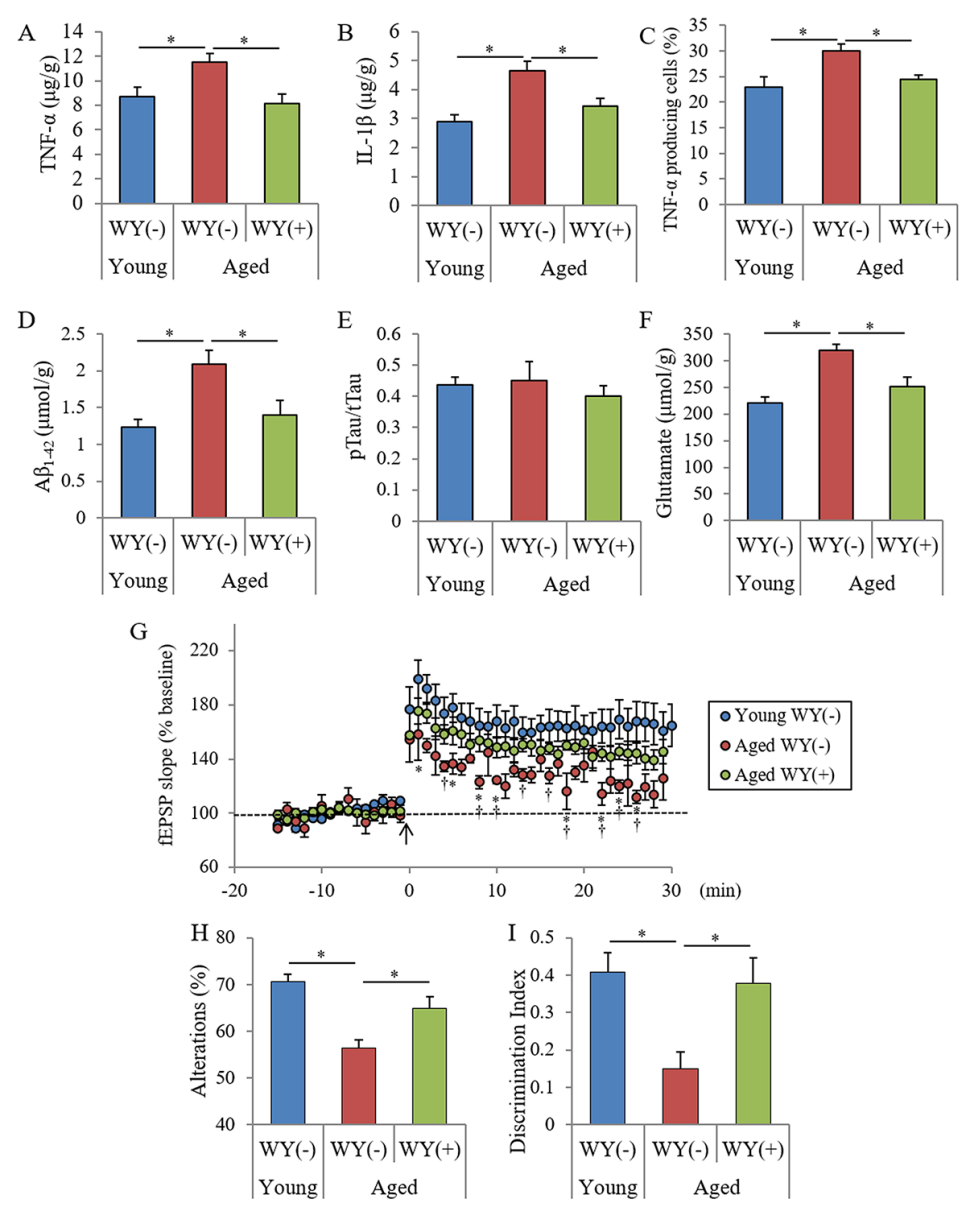
**Effects of WY peptides on cognitive decline in aged mice.** Seven and 68-week-old male C57BL/6J mice were maintained on a diet with or without 0.05% (w/w) WY peptide for 19 weeks. (**A, B**) The levels of TNF-α and IL-1β in the hippocampus. (**C**) Characterization of CD11b-positive microglia in the brain isolated by MACS and flow cytometry. The percentages of TNF-α-producing cells to CD11b-positive cells. (**D-F**) The levels of TBS-soluble Aβ_1-42_, pTau (pS199)/total Tau, and glutamate in the hippocampus. (**G**) LTP measurement from a hippocampus slice after theta-burst stimulation (arrow). (**H**) Spontaneous alterations during 8 min of exploring in Y-maze. (**I**) Discrimination index in NORT by measuring the time spent exploring novel and familiar objects during 5 min of re-exploration. Data are the mean ± SEM of 7-week-old mice (n = 15) fed with control diet, 68-week-old mice (n = 13) fed with control diet, and 68-week-old mice (n = 10) fed with diet containing WY peptide. *p*-values shown in the graph were calculated by one-way ANOVA followed by the Tukey–-Kramer test. *p*-values shown in the graph were calculated by unpaired *t*-test in (G). *p < 0.05,†*p* < 0.05.

Following the evaluation showing that the WY peptide suppressed inflammatory responses against LPS stimulation in the brain, we sought to examine the effects of the WY peptide on chronic inflammation in the brain associated with aging. Seven- or 68-week-old mice were fed a diet containing the 0.05% (w/w) WY peptide and then cognitive functions were measured. The amounts of TNF-α and IL-1β in the hippocampus of aged mice fed the control diet were significantly increased compared with those of young mice, and the amounts of TNF-α and IL-1β were significantly decreased in aged mice fed the WY peptide-containing diet compared with aged mice fed the control diet (Figures 4A and B, respectively). The ratio of TNF-α–producing cells in CD11b-positive microglia was also increased in the aged mice compared with the ratio in young mice, and the ratio in aged mice fed WY peptide-containing diet decreased compared with aged mice fed the control diet (Figure 4C). These results indicate that inflammation in the hippocampus of the aged mice was associated with aging and that the intake of the WY peptide suppressed the activation of the microglia and inflammation.

Next, to examine the levels of neurotoxin production and agents inducing inflammation, the amounts of Aβ, phosphorylated Tau, and glutamate were measured. The amounts of Tris-buffered saline (TBS)-soluble Aβ_1-42_ and glutamate in the hippocampus of aged mice increased relative to those in young mice, and the amounts in the aged mice fed with WY peptide-containing diet decreased relative to those in the aged mice fed the control diet (Figures 4D and F). However, the ratio of phosphorylated Tau to total Tau did not change between the young and aged mice (Figure 4E). In the frontal cortex, the levels of Aβ_1-42_ and glutamate in aged mice were also higher than those in the young mice and were reduced following administration of the WY peptide (data not shown). These results indicated that Aβ_1-42_ and glutamate in the hippocampus were increased with aging and were attenuated by the administration of WY peptide.

To investigate whether WY peptide treatment affected synaptic plasticity, we evaluated the magnitude of long-term potentiation (LTP) in the Schaffer collateral-CA1 pathways in acute hippocampal slices. Previous studies have demonstrated that aging and neuroinflammation impair LTP in the Schaffer collateral-CA1 pathways in the hippocampus, which plays an important role in memory and learning [22]. Therefore, we evaluated whether intake of the WY peptide could rescue the magnitude of the LTP deficit induced by aging observed in hippocampal slices from aged mice. As shown in Figure 4G, the magnitude of LTP induced by theta-burst stimulation in aged control mice without the WY peptide was significantly reduced relative to that in the young mice. On the contrary, the suppressed LTP in aged mice was reversed by treatment with the WY peptide.

To evaluate the effects of the WY peptide on memory impairment in aged mice, spatial memory and object recognition memory were measured. The spontaneous alterations and discrimination index of NORT were also decreased in aged mice relative to those in young mice, and those in aged mice fed with the diet containing the WY peptide were significantly increased relative to those in aged mice fed with control diet (Figures 4H and I, respectively). In addition, the spontaneous alterations and the discrimination index in aged mice orally administered 10 mg/kg WY peptide for 14 days was significantly increased compared with those in control aged mice (Figures S1A and S1B, respectively). These results indicated that intake of the WY peptide prevented and improved memory impairment in aged mice.

Taken together, the results indicated that the intake of the WY peptide suppresses inflammation in the brain and prevents deficits in neuronal activity and memory impairment associated with aging.

### Preventive effects of the WY peptide on Alzheimer’s pathology and cognitive decline

In aged mice, intake of the WY peptide reduced the levels of Aβ and inflammatory cytokines in the hippocampus and prevented memory impairment. To evaluate the effects of the WY peptide on the activation of microglia and pathology in Alzheimer’s disease, 5×FAD mice were fed with the 0.05% (w/w) WY peptide, and inflammation and Aβ pathology and memory functions were examined.

**Figure 5:**
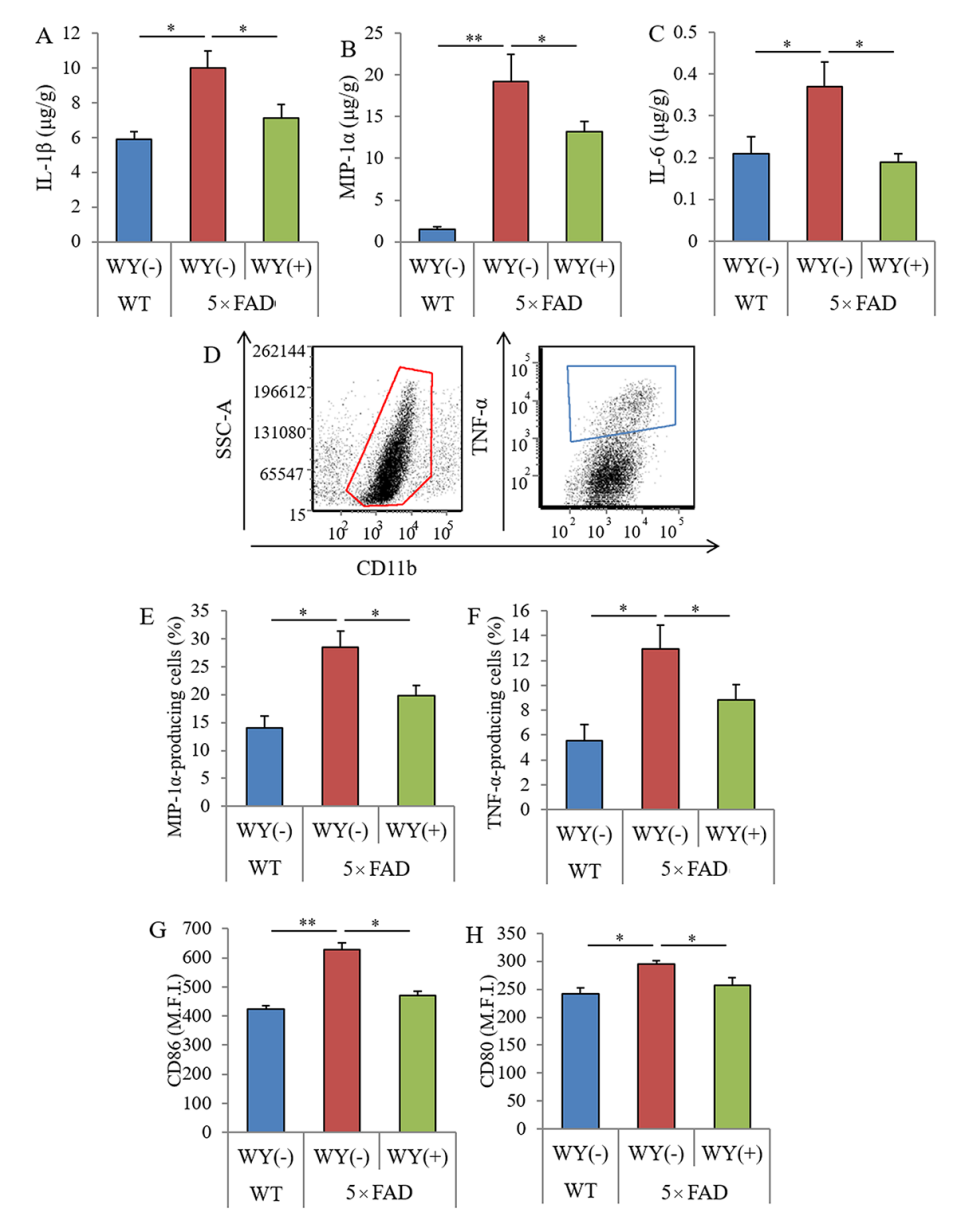
**Effects of WY peptides on inflammation and the activation of microglia in 5×FAD mice.** For 3 months, 2.5-month-old transgenic 5×FAD and wild-type male mice were fed a diet with or without 0.05% w/w WY peptide. (**A–C**) The levels of IL-1β, MIP-1α, and IL-6 in the hippocampus, respectively. Data represent the means ± SEM of 12 wild-type mice, 11 control transgenic mice, and 12 transgenic mice fed with the diet containing WY peptide. (**D**) Characterization of CD11b-positive microglia in the brain isolated with MACS by flow cytometry. (**E, F**) Ratio of MIP-1α- and TNF-α-producing cells to CD11b-positive cells, respectively. (**G, H**) Expressions of CD80 and CD86 on CD11b-positive cells, respectively. M.F.I. is the mean fluorescent intensity. Data represent the means ± SEMs of 6 mice per group. *p*-values shown in the graph were calculated by one-way ANOVA followed by the Tukey–Kramer test. *p < 0.05, **p < 0.01.

**Figure 6:**
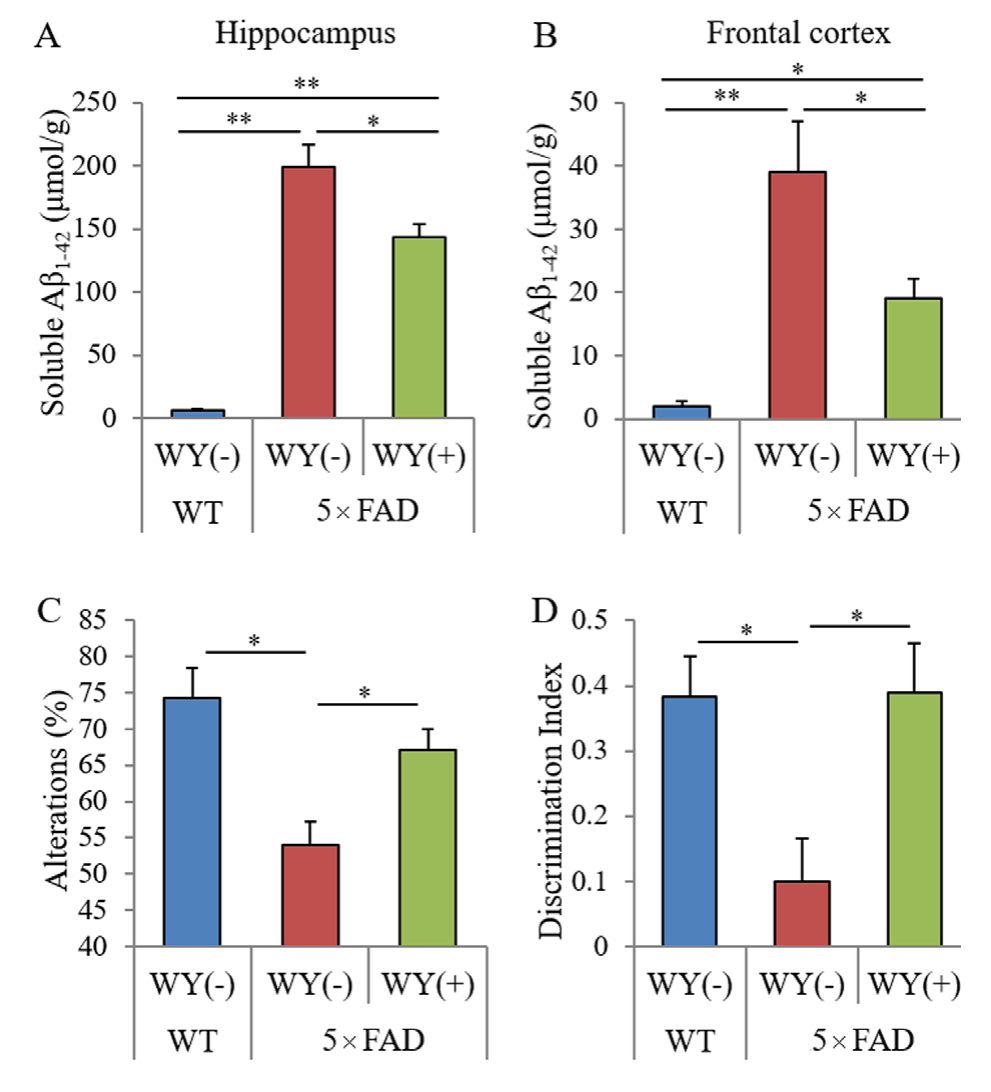
**Effects of WY peptide on Aβ deposition and memory function in 5×FAD mice.** For 3 months, 2.5-month-old transgenic 5×FAD and wild-type male mice were fed a diet with or without 0.05% w/w WY peptide. (**A, B**) The levels of TBS-soluble Aβ_1-42_ in the hippocampus and frontal cortex, respectively. Data represent the means ± SEM of 12 wild-type mice, 11 control transgenic mice, and 12 transgenic mice fed with a diet containing the WY peptide. Data represent the means ± SEM of 7 mice per group. (**C**) Spontaneous alterations during 8 min of exploring in Y-maze. (**D**) Discrimination index in NORT by measuring time spent exploring novel and familiar objects during 5 min of re-exploration. *p*-values shown in the graph were calculated by one-way ANOVA followed by the Tukey–Kramer test. *p < 0.05.

The levels of IL-1β, MIP-1α, and IL-6 in the hippocampi of the 5×FAD mice were significantly higher than those of the wild-type mice. The levels of these cytokines in 5×FAD mice fed the WY-containing diet were significantly lower than those in 5×FAD control mice fed the control diet (Figures 5A–C, respectively). In addition, CD11b-positive microglia in the brain were isolated, and cytokine production and the expressions of stimulatory molecules were measured by flow cytometry (Figure 5D). The percentages of MIP-1α and TNF-α-producing CD11b-positive microglia and the expressions of CD86 and CD80 on the microglia were significantly increased in the brains of 5×FAD mice compared with those of wild-type mice. The percentages and expressions of these cytokines were significantly decreased in 5×FAD mice fed a WY peptide-containing diet compared with those in 5×FAD control mice (Figure 6E–H, respectively). These results show that the WY peptide suppresses inflammation and activation of the microglia in the brains of 5×FAD mice.

Next, to evaluate the effects of the WY peptide on Aβ pathology, the levels and deposition of Aβ were measured semi-quantitatively. The levels of TBS-soluble Aβ in the hippocampi and frontal cortexes of 5×FAD mice fed WY peptide-containing diet was significantly reduced compared with those of 5×FAD control mice (Figure 6A and B, respectively). Immunohistochemical analysis for Aβ deposition (Supplementary Figure S2A and B) showed that Aβ deposition in the olfactory and visual cortexes of the 5×FAD fed WY peptide-containing diet was significantly reduced compared with that of 5×FAD control mice (Supplementary Figure S2C). Immunohistochemical analysis for Iba-1 of activate microglia (Supplementary Figure S2D) showed that Iba-1-positive microglia in the olfactory cortexes of the 5×FAD fed WY peptide-containing diet was significantly reduced compared with that of 5×FAD control mice. These results indicate that the intake of the WY peptide suppresses Aβ deposition and the activation of microglia in the brain.

Last, to investigate the effects of the WY peptide on memory impairment, 5×FAD mice administered the WY peptide were subjected to the Y-maze test and NORT. The spontaneous alterations of the Y-maze test and discrimination index of the NORT in 5×FAD mice were decreased compared with those in wild-type mice. Those alternations were significantly increased in 5×FAD mice fed WY peptide-containing diet compared with those in 5×FAD control mice (Figure 6C and D, respectively). These results indicate that intake of the WY peptide prevents memory impairment in 5×FAD mice.

## DISCUSSION

In this study, we originally found that the WY and WM dipeptides, which contain tryptophan in the N-terminal, suppressed the microglial activation against LPS and enhanced microglial phagocytosis of Aβ. Intake of the WY peptide suppressed microglial activation and prevented memory impairment in LPS-injected mice, aged mice, and Alzheimer’s mice model. These results suggest that oral consumption of the WY peptide suppresses the microglial activation in the brain triggered by various inflammatory stimulations. A previous study demonstrated that dipeptides that include tryptophan in the N-terminal had the highest affinity to the peptide transporter, and the WY peptide is one of the peptides with the highest affinities to the peptide transporter [23]. According to this report and the pharmacokinetics in the present study, orally administered WY peptide is supposed to be absorbed more smoothly into the body than are other dipeptides and is delivered to the brain where it suppresses microglial activation. It has recently been reported that orally administered β-lactopeptide of glycine–threonine–tryptophan–tyrosine (GTWY), β-lactolin, and its core sequences of WY peptide inhibit the activity of monoamine oxidase B (MAO-B) in the brain [24]. It has also has been reported that the MAO-B inhibitor reduces reactive oxygen species and suppresses Parkinson’s disease pathology [25]. In addition, it has been demonstrated that danshensu, which has an inhibitory activity of MAO-B, reduces the activation of nuclear factor kappa B (NF-κB) and suppresses NF-κB-regulated pro-inflammatory responses [26]. Taken together, the results suggest that following oral administration of the WY peptide, it is delivered to the brain and inhibits MAO-B activity, resulting in the suppression of inflammatory responses in the brain. However, we did not evaluate the involvement of MAO-B activity with the disease pathology in the 5xFAD mice, so further study need to examine the effects of WY peptide on MAO-B activity in the brain of 5xFAD mice. In addition, the present study did not evaluate the effects of WY peptide on the groups of young mice and wild-type mice, so further study need to examine the effects on these groups.

LPS is known to be an agonist for toll-like receptor 4, which induces pro-inflammatory cytokine production in the microglia. It has been reported that LPS treatment leads to the reduction of dendritic spine densities and inhibition of LTP in the hippocampus [27]. For instance, docosahexaenoic acid suppressed the microglial activation, which resulted in the restoration of synaptic structures and functions in hippocampal CA1 pyramidal neurons [28]. Pyramidal neurons in the CA1 region of the hippocampus are crucial for spatial memory and object recognition memory. Experiments using CA1-specific N-methyl-D-aspartate receptor 1 subunit-knockout mice have shown that neuronal activation in CA1 is crucial for spatial memory and experience-induced synaptic plasticity [29,30]. Intake of the WY peptide has been shown to attenuate LPS-induced atrophy of dendritic spine densities. Regarding LTP, several studies have shown that inflammation and activation of microglia are associated with aging [31] and are associated with suppression of LTP [32]. In particular, it was demonstrated that IL-1β and TNF-α inhibited LTP *in vitro* [32,33], and treatment with minocycline suppressed pro-inflammatory cytokines in microglia and restored LTP in aged rats and primary culture [32,34]. Intake of the WY peptide restored the LTP suppression in the CA1 region of the hippocampus in aged mice. These reports suggest that the suppression of inflammation through WY peptide intake prevents the decrease in synaptic plasticity in the hippocampus, leading to the reduction in cognitive decline. These reports suggest that microglial inflammation is associated with dysfunction of neurons in CA1 and impairments depending on the hippocampus, and that the suppression of microglial activation is fundamental for preventing memory impairment in aged and 5×FAD mice by the consumption of the WY peptide.

In 5×FAD mice, intake of the WY peptide also reduced the level of Aβ and suppressed inflammation in the brain. It is suggested that microglial phagocytosis of Aβ enhanced by the WY peptide reduced the level of Aβ in the hippocampus. On the contrary, the involvement of the WY peptide on the cleavage of the Aβ precursor protein (APP) and expression of APP were not evaluated [35]. Therefore, further studies are needed to elucidate the underlying mechanism. In 5×FAD mice, intake of the WY peptide suppressed microglial activation in the brain. Activated microglia exacerbates the disease pathology of Alzheimer’s disease via inflammatory responses; hence, regulation of microglial activation by the WY peptide shows preventive effects on Alzheimer’s pathology.

It has been reported that inflammation in the brain accelerates cognitive decline and dementia pathology. Therefore, prevention of inflammation in the brain is a therapeutic target for preventing dementia and other neurological disorders. Non-steroidal inflammatory drugs are under evaluation for the prevention of Alzheimer’s disease [36,37], but medicinal treatments have a risk of side/adverse effects. Our study was based on epidemiological and clinical studies that have shown prevention of cognitive decline and dementia following the intake of dairy products [14]. In addition, our group has previously demonstrated that Camembert cheese and fermented dairy products with fungi suppress inflammation and Alzheimer’s pathology in the 5×FAD mice [13]. However, previous reports have not clearly identified the responsible agents in dairy products that prevent Alzheimer’s pathology. Our study results support these previous epidemiological, clinical, and preclinical study results. A recent study reported that consumption of whey peptide rich in the WY-related β-lactolin improves cognitive performance in elderly individuals with subjective cognitive decline [38]. It has been reported that subjects with mild cognitive impairment showed brain inflammation that accompanied Aβ accumulation [39]. Therefore, the suppression of inflammation in the brain using the WY peptide might be associated with memory improvement. Further studies are warranted to elucidate the relationship between memory improvement by the WY peptide and the activation of microglia clinically. The WY peptide derived from whey peptides, which are easy and safe to ingest in daily life, might provide a new approach for the prevention of memory impairment related to neuronal inflammation.

## MATERIALS AND METHODS

### Animals

Newborn (<7-day-old), 7-week-old male, and 68-week-old male C57BL/6J mice and 6-week-old male ICR mice (Charles River Japan, Tokyo, Japan) were maintained at the Kirin Company Ltd. All experiments were approved by the Animal Experiment Committee of Kirin Company Ltd., and were conducted in 2015 in strict accordance with their guidelines. All efforts were made to minimize suffering.

Alzheimer’s disease model mice, B6SJL-Tg mice [APPSwFlLon, PSEN1*M146L*L286V, http://jaxmice.jax.org/strain/006554.htmL [40]], hereafter referred to as 5×FAD transgenic mice, were purchased from Jackson Laboratory (Sacramento, CA, USA) and maintained by crossing hemizygous transgenic mice with B6SJLF1/J mice in the experimental facility at the University of Tokyo. 5×FAD transgenic mice overexpress mutant human APP (695) with the Swedish (K670N, M671L), Florida (I716V), and London (V717I) Familial Alzheimer's Disease (FAD) mutations, along with human PS1 harboring 2 FAD mutations, M146L and L286V. Nontransgenic wild-type littermates were used in all experiments. All experiments were approved by the Animal Care and Use Committee of the Graduate School of Agricultural and Life Sciences, the University of Tokyo, and were conducted the experiments in 2016 in strict accordance with their guidelines. All efforts were made to minimize suffering.

Mice were allocated into each group without significant differences about weight among the groups. Mice <3 months of age were fed a standard purified rodent growth diet (AIN-93G; Oriental Yeast, Tokyo, Japan), and those ≥3 months of age were fed a maintenance diet (AIN-93M; Oriental Yeast).

SD rats aged 7 weeks (Charles River Japan) were maintained at Sekisui Medical Ltd. The rats were fed a certified diet (MF; Oriental Yeast). The experiments were approved by the Animal Experiment Committee of Sekisui Medical Ltd. and conducted in 2016 in strict accordance with their guidelines.

### Preparation of peptide samples from casein and analysis of the peptides

To prepare the peptide products, 5% (w/v) casein sodium (Wako, Osaka, Japan) was dissolved in 0.05 M Tris-buffer (pH 7.5) and digested by 0.125% (w/v) enzymes from *Aspergillus melleus* (Protease P 「amano」 3SD; Amano Enzyme Inc., Aichi, Japan) at 50°C for 4 h, as performed and described in our previous report [24]. After the enzyme reaction, the samples underwent ultrafiltration through a 10-kDa membrane to remove the undigested proteins and enzymes. The products were used for the assay.

Digested products were fractionated by using solid-phase columns. Digested products were dissolved in water and applied to a Sep-Pak C18 cartridge column (Nihon Waters, Tokyo, Japan). Bound compounds were eluted with 20%, 40%, 60%, 80%, and 100% methanol. Further separations were performed using high-performance liquid chromatography (HPLC; Shimadzu, Kyoto, Japan). Samples were separated on a 4.6 × 250-mm^2^ Aeris peptide 3.6-μm column (Shimadzu) at 70°C by using a flow rate of 1.0 mL/min. The separation protocol was as follows: linear gradient from 95% water-trifluoroacetic acid (100:0.1, v/v)/5% acetonitrile-trifluoroacetic acid (100:0.1, v/v) gradually to 70%/30% over 30 min. Peaks at 210 nm were detected by using an SPD-M20A photodiode detection system (Shimadzu).

The sequences of purified peaks showing the activities were analyzed using the Edman sequencing method. Each sequenced peptide was synthesized (Eurofins, Tokyo, Japan) and identified as the same peak purified and sequenced, and then the activity was evaluated again. A massive amount of tryptophan–tyrosine (WY) and tryptophan–methionine (WM) peptides were purchased from Bachem (Bubendorf, Switzerland).

### *In vitro* microglial assay

Evaluation using primary microglia for anti-inflammatory and phagocytic activities has been previously described [41]. Briefly, primary microglial cells were isolated from mouse brain via magnetic cell sorting (MACS) after conjugation with anti-CD11b antibodies. Isolated CD11b-positive cells (of >90% purity as evaluated by flow cytometry) were plated in poly-D-lysine (PDL)-coated 96-well plates (BD Biosciences, Billerica, MA, USA) and cultured in DMEM/F-12 (Gibco, Carlsbad, CA, USA) medium supplemented with 10% fetal calf serum (Gibco) and 100 U/mL penicillin/streptomycin (Sigma-Aldrich, St Louis, MO, USA).

For the assay of anti-inflammatory activity, isolated microglia were plated at a density of 30,000 microglia/well on a PDL-coated 96-well plate, treated with each sample for 12 h, and then treated with lipopolysaccharide (LPS, 5 ng/mL, Sigma-Aldrich, St. Louis, MO, USA) and interferon-γ (0.5 ng/mL, R&D systems, Minneapolis, MN, USA) for 12 h. After stimulation, supernatants were applied to a TNF-α production assay. To quantify TNF-α, the supernatant was measured by using an enzyme-linked immunosorbent assay (ELISA) kit (eBiosciences, San Diego, CA, USA). For measuring intracellular cytokine production, microglia were treated with a leukocyte activation cocktail with BD GolgiPlug (BD Biosciences) and analyzed by flow cytometry after staining with anti-mouse CD11b-APC-Cy7 (M1/70, BD Pharmingen, San Diego, CA, USA), anti-mouse TNF-α-FITC (MP6-XT22, BD Pharmingen), and anti-mouse MIP-1α-PE (DNT3CC; eBiosciences) antibodies and the Cytofix/Cytoperm Fixation/Permeabilization kit (BD Biosciences). Expression of cell-surface markers were analyzed by flow cytometry after staining with anti-mouse CD11b-APC-Cy7 (M1/70, BD Pharmingen), anti-mouse CD80-APC (16-10A1; eBiosciences), and anti-mouse CD86-PE (GL1; eBiosciences) antibodies.

For the assay of phagocytic activity, microglia were plated at a density of 50,000 cells/well on PDL-coated 96-well plates and incubated with 500 nM 6-carboxyfluorescein-labeled Aβ_1-42_ (Aβ-FAM; AnaSpec, Fremont, CA, USA) for 24 h after sample treatment for 12 h. After the medium was removed, extracellular Aβ-FAM was quenched with 0.2% trypan blue, pH 4.4. The cellular fluorescence intensity of the entire areas of the 5 wells/sample was measured at 485 nm excitation/535 nm emission by using a plate reader (Molecular Device, Sunnyvale, CA, USA).

### Pharmacokinetics of dipeptide

^14^C-labeled WY, Trp-[carboxyl-^14^C]Tyr (^14^C-WY), was prepared by Sekisui Medical Ltd. The radiochemical purity assessed by HPLC was 99.3%, and its specific radioactivity was 5.76 MBq/mg. The test solution ^14^C-WY (0.1 mg/mL) was dissolved in distilled water and prepared just before its administration. SD rats (n = 3) were starved for 16 h and administered the solution orally (10 mL/kg body weight, 5.76 MBq/kg). At 2, 5, 10, and 30 min and at 1, 2, 4, 6, 8, 10, 24, 48, and 72 h after administration, the radioactivities of the plasma samples were quantified by using a liquid scintillation counter (HIONIC-FLUOR; PerkinElmer, MA, USA) At 2 h after administration, the radioactivity of each organ homogenate was quantified by using the scintillation counter.

### Neuroinflammatory model mice injected intracerebroventricularly with LPS

To evaluate the effects of the WY peptide on inflammation in the brain, 6-week-old ICR male mice (n = 10 per group) were orally administered 0, 3, or 30 mg/kg of the WY peptide dissolved in distilled water, once a day for 8 days. Thirty minutes after the last administration, the mice were deeply anesthetized with sodium pentobarbital (Kyoritsu Seiyaku, Tokyo, Japan) and injected intracerebroventricularly with 5 μg of LPS (L4391; Sigma) to induce the inflammation in the brain. Four hours later, the mice were euthanized and their brains were removed, as shown in Figure 2A. The left hippocampus and frontal cortex were homogenized in Tris-buffered saline (TBS) buffer (Wako) containing protease inhibitor cocktail (Biovision) with a multi-beads shocker (Yasui Kikai, Osaka, Japan). After centrifugation at 50,000 × g for 20 min, the supernatant was collected. The total protein concentration of each supernatant was measured using a BCA protein assay kit (ThermoScientific, Yokohama, Japan). To quantify the cytokine and chemokine levels in the brain, the concentrations of TNF-α (eBioscience), IL-1β (eBiosciences), and macrophage inflammatory protein 1α (MIP-1α) (R&D Systems) in the supernatants were measured by ELISA. The right hemisphere was used for morphological analysis of the dendrites. Brain sections at the bregma −2.06 mm were prepared and stained by using the FD Rapid GolgiStain Kit (FD Neuro Technologies, MD, USA) according to the manufacturer’s instructions. Spines were counted within the CA1, and the prefrontal cortex dendrites were counted starting from their point of origin from the primary dendrite, as previously described. For spine density measurements, all areas containing 50–100 μm of secondary dendrites from the neuron were used. In the other experiment to evaluate cognitive function, mice were injected intracerebroventricularly with 15 μg of LPS, which was continuously administered daily with each sample. Spontaneous alterations by the Y-maze test were performed at 3 days post-injection, and the novel-object recognition test (NORT) was performed at 4 and 5 days post-injection, as described in the following sections.

### Aged mice displaying chronic inflammation associated with aging

To evaluate the effects of the WY peptide on inflammation and cognitive decline in aged mice, 7-week-old male and 68-week-old male C57BL/6J mice (n = 15 in 7-week-old control mice, n = 13 in 68-week-old control mice, n = 10 in 68-week-old mice administered the WY peptide) were maintained with a diet containing 0% or 0.05% (w/w) WY peptide for 19 weeks. At 16 weeks after administration, the spontaneous alteration test and NORT were performed. At 19 weeks after administration, the mice were sacrificed and the concentration of TNF-α (eBiosciences), IL-1β (eBiosciences), Aβ_1-42_ (Wako), total Tau (Thermo Scientific), and pTau (pS199, Thermo Scientific) were measured after the same preparation as described in the previous section. To evaluate the effects of oral administration of the WY peptide for a few weeks on cognitive function in aged mice, 23- and 84-week-old male C57BL/6J male mice (n = 10 in 23-week-old control mice, n = 10 in 84-week-old control mice, and n = 9 in 84-week-old mice administered the WY peptide) were administered 0 or 10 mg/kg WY peptide for 14 days and subjected to behavioral evaluations, the Y-maze test, and the NORT. To evaluate the levels of glutamate in the brain, tissue was homogenized in methanol (Wako). After centrifugation and filtration, the samples were derivatized with o-phthaldialdehyde/2-mercaptoethanol (Wako) and analyzed by HPLC using an EICOMPAK FA-3ODS column and CA-ODS precolumn (Eicom, Kyoto, Japan) with electrochemical detection. The mobile phases consisted of 100 mM phosphate buffer (pH 6.0) containing 7% (v/v) methanol (Wako) and 13% acetonitrile (Wako), and 100 mM phosphate buffer (pH 6.0) containing 50% acetonitrile. To analyze the microglial activity in the brain, CD11b microglia were isolated by using MACS, and intracellular cytokines in the microglia were measured by flow cytometry, as described in the previous section. To evaluate the neuronal activity in the hippocampus, LTP using hippocampal slices were measured, as described in the following section.

### Electrophysiology for long-term potentiation measurement

Acute hippocampal slices of 300-μm thickness were obtained from 7- and 68-week-old male mice in artificial cerebrospinal fluid (aCSF) that contained 10 mM D-glucose, 3 mM KCl, 26 mM NaHCO_3_, 124 mM NaCl, 3 mM KCl, 2 mM CaCl_2_, 1 mM MgSO_4_, and 1.25 mM KH_2_PO_4_. The aCSF was saturated with 95% O_2_/5% CO_2_ throughout the experiments. The slices were then maintained at 32°C for ≥45 min in aCSF for recovery before recordings. Recordings were performed by using the Med64-Quad II multielectrode array system (Alpha MED Scientific) in the CA1 region following stimulation of the Schaffer collateral path while refluxing with aCSF (1.5–2.0 mL/min). Field Excitatory Post Synaptic Potentials (fEPSP) were elicited and recorded via planar electrodes of the Quad II 2×8 Probe AL-MED-PG501A by aligning the electrodes and the stratum radiatum region of hippocampal slices. An input–output curve was performed at the beginning of each recording to determine the appropriate stimulation intensity. Test stimuli at 30%–40% of maximal intensity were delivered at 0.05 Hz, and a stable baseline of fEPSP of ≥15 min was established before LTP induction. LTP was induced by applying patterned theta-burst stimuli via the stimulation electrode. Theta-burst stimuli comprised triple stimuli at 50 Hz 3 times at 5 Hz, and the stimulus intensity was set to 50%–60% of the maximum intensity. Recordings were performed for 30 min after LTP induction. Data analysis was performed by using Med64 Mobius Software (Alpha MED Scientific).

### 5×FAD Alzheimer’s model mice exhibiting neuroinflammation induced by Aβ deposition

To evaluate the effects of the WY peptide on Alzheimer’s-like disease, 2.5-month-old transgenic 5×FAD and wild-type male mice were fed a diet with or without 0.05% w/w WY peptide for 3 months (n = 12 in wild-type mice, n = 11 in transgenic control mice, n = 12 in transgenic mice given the WY peptide). The mice were then euthanized and their brains were removed. The TBS-soluble homogenate of the left hippocampus and frontal cortex were prepared as described in the previous section. The pellets were homogenized again in TBS containing 1% Triton X-100 (Wako), and the supernatant was collected after centrifugation. The first supernatant was assayed for quantifying soluble Aβ_1-42_ (Wako) by ELISA and cytokines and chemokines by a Bio-Plex assay system (Bio-Rad, Hercules, CA, USA). The second supernatant was used for quantifying insoluble Aβ_1-42_ (Wako) by ELISA. To evaluate Aβ deposition and microglial infiltration immunohistochemically, the right brain hemispheres (n = 6–7 in each group) were fixed in 10% formalin solution (Wako), paraffin-embedded, and cut into 5-µm sections. Each brain region, including the hippocampus and cerebral cortex (bregma 2.30 mm posterior), were analyzed with a monoclonal anti-human Aβ_x-42_ antibody (12F4; Millipore, Billerica, MA, USA). The size of the positive region per area was measured by using Image J image analysis software (NIH, Bethesda, MD, USA). To evaluate microglia activity, the right brain hemispheres (n = 5 in each group) were removed, and the isolated CD11b-positive microglia were used for the phagocytic assay and flow cytometric analysis, as described previously. To evaluate the cognitive function, mice were subjected to the Y-maze test and NORT.

### Spontaneous alteration test

The Y-maze is a three-arm maze with equal angles between all arms (25-cm long × 5-cm wide × 20-cm high). The maze walls were constructed from dark black polyvinyl plastic. Each mouse was initially placed in 1 arm, and the sequence and number of arm entries were counted for 8 min. The alternation score (%) for each mouse was defined as the ratio of the actual number of alternations to the possible number (defined as the total number of arm entries minus two) multiplied by 100 as follows:

% Alternation = [(Number of alternations) / (Total arm entries − 2)] × 100.

### Novel-object recognition test

A NORT was performed during the light period in a polyvinyl chloride box (25 × 40 × 20 cm^3^) without a roof. For the acquisition trial, a pair of wooden triangle poles (4.5 × 4.5 × 4.5 cm^3^) or wooden pyramids (4.5 × 4.5 × 4.5 cm^3^) was used; for the retention trial, a pair of poles or pyramids and a golf ball (4.5-cm diameter) were used. In all trials, the objects were placed 7.5 cm apart from the corner of the box. In the acquisition trial, each mouse was allowed to explore the box with the 2 objects for 10 min. Twenty-four hours after the acquisition trial, the mouse was allowed to explore the box with the novel and familiar objects for 5 min. The discrimination index was calculated by dividing the difference in the time for exploring the novel object and for the familiar object by the total time spent exploring both objects: that is, (novel object exploration time – familiar object exploration time)/(total exploration time); thus, a discrimination index of 0 indicated equal exploration of both objects.

### Statistical analysis

The data are presented as the mean ± SEM. Data were analyzed by using one-way ANOVA followed by the Tukey–Kramer, Dunnett, and Student *t*-tests, as described in the figure legends. All statistical analyses were performed using the Ekuseru-Toukei 2012 software program (Social Survey Research Information, Tokyo, Japan).

## SUPPLEMENTARY MATERIAL

Supplementary Figures
