# Supplementary material for: Tryptophan-related dipeptides in fermented dairy products suppress microglial activation and prevent cognitive decline
Source: Aging (Albany NY). 2019 May 23;11(10):2949–67. doi: 10.18632/aging.101909 (PMC6555451; doi:10.18632/aging.101909)
Supplement: Supplementary Figures [file aging-11-101909-s001.pdf]

SUPPLEMENTARY MATERIAL

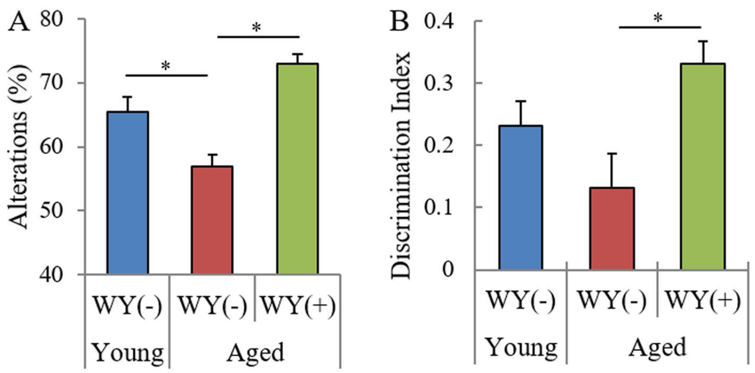

Supplementary Figure S1. Effects of short-term administration of the WY peptide on memory impairment in aged mice. For 14 days, 23- and 84-week-old male C57Bl/6J male mice were administered 0 or 10 mg/kg WY peptide. (**A, B**) Spontaneous alterations in Y-maze and discrimination index in NORT, respectively. Data are the means ± SEM of 23-week-old mice (n = 10), 84-week-old mice (10) given 0 mg/kg WY peptide, and 84-week-old mice (n = 9) given 10 mg/kg WY peptide.

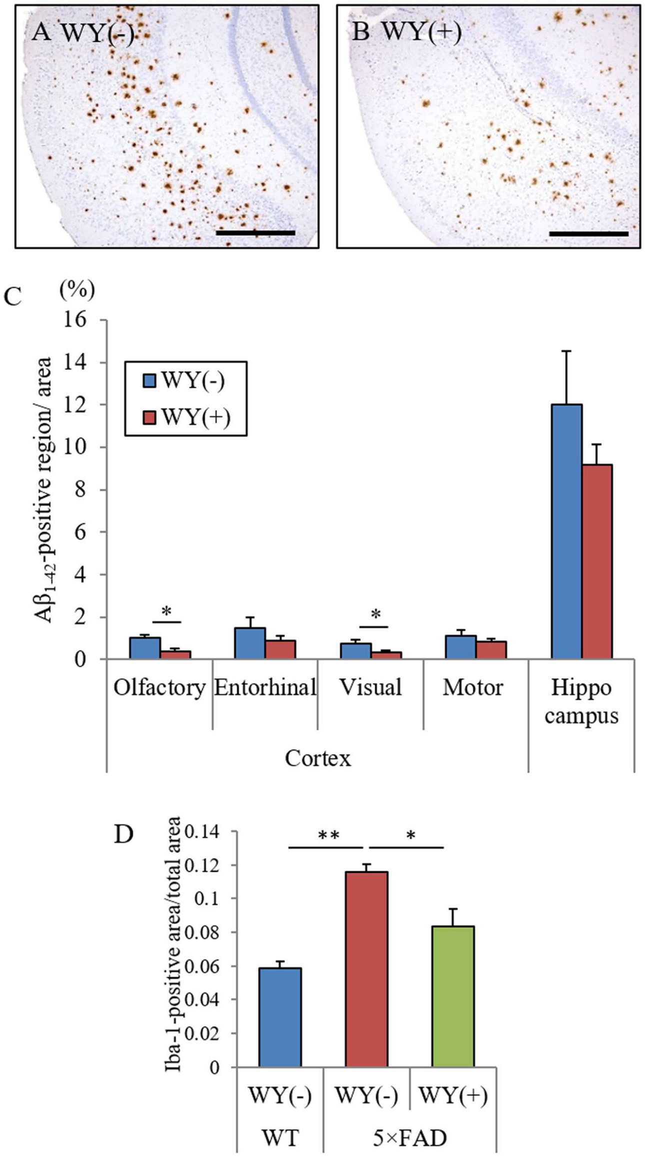

Supplementary Figure S2. Effects of WY peptide on Aβ deposition in 5×FAD mice. Percent positive area for $A\beta_{1-42}$ in the brain of the transgenic control mice and transgenic mice fed with a diet containing WY peptide. (**A, B**) The representative images of immunohistochemistry for $A\beta_{1-42}$ in transgenic mice with or without 0.05% WY peptide, respectively. (**C**) Semiquantification for $A\beta_{1-42}$ detected immunohistochemically in each brain region. (**D**) Semiquantification for Iba-1 detected immunohistochemically in olfactory cortex. Data represent the means ± SEM of 7 mice per group. *p*-values shown in the graph were calculated by performing Student *t*-test. $^*p < 0.05$.
